# Scalability of Wi-Fi Performance in Virtual Reality Scenarios

**DOI:** 10.3390/s25206338

**Published:** 2025-10-14

**Authors:** Vyacheslav Loginov, Sergei Tutelian, Ivan Startsev, Evgeny Khorov

**Affiliations:** Wireless Networks Lab, Institute for Information Transmission Problems of the Russian Academy of Sciences, Moscow 127051, Russia; tutelian@wnlab.ru (S.T.); startsev@wnlab.ru (I.S.); khorov@wnlab.ru (E.K.)

**Keywords:** Virtual Reality, Metaverse, Wi-Fi, scheduling, MU-MIMO

## Abstract

The adoption of Virtual Reality (VR) applications in Wi-Fi networks intensifies each year. VR applications impose strict Quality of Service (QoS) requirements, necessitating low latency and high throughput. Meeting VR QoS requirements in Wi-Fi networks is especially challenging due to unpredictable channel fading and interference. This paper presents a comprehensive scalability study of Wi-Fi performance in multi-user VR scenarios. We investigate whether simply increasing Access Point (AP) capabilities, specifically through Multi-User MIMO (MU-MIMO), is sufficient to support dense VR deployments. To this end, we developed a high-fidelity simulation framework in ns-3 to estimate the network capacity when serving VR traffic. Our analysis meticulously evaluates the impact of critical factors, including the number of antennas at the AP and STAs, MU-MIMO scheduling algorithms, channel sounding period, and different channel conditions. The results reveal a critical finding: Scalability is not linear. In particular, doubling AP antennas from 8 to 16 yields only a 35% gain in capacity under typical conditions, not the 100% linear scaling one might expect. We identify and analyze the key bottlenecks that prevent performance from scaling indefinitely with an increased number of AP antennas, providing crucial insights for the design of next-generation Wi-Fi systems aimed at supporting the Metaverse and future immersive VR applications.

## 1. Introduction

A noticeable trend today is the increasing need for Metaverse and Virtual Reality (VR) applications. These VR applications have emerged recently, spanning fields like entertainment [1], medicine [2], engineering [3], and different aspects of our daily lives. They impose strict Quality of Service (QoS) requirements according to which all frames shall be delivered from the VR server to the VR head-mounted display (HMD) of the user within a fixed delay bound, e.g., within 20 ms with probability higher than 99% [4,5,6]. Obviously, the truly immersive VR experience is only possible in the case of wireless HMD, and off-the-shelf VR devices mainly use Wi-Fi for wireless connection to the network [7]. However, because of unpredictable channel contention and fading, the problem of serving VR traffic is becoming especially complicated in Wi-Fi networks.

The challenge of VR QoS provisioning can be addressed through various methods implemented at the Wi-Fi access point (AP). In particular, in this paper, we investigate the scalability of the two main strategies for improving VR performance in Wi-Fi networks:

1. The increase in the number of AP antennas to enhance data rates with the multiple-input multiple-output (MIMO) technology.
2. QoS-aware transmission schedulers that take into account delays of currently enqueued frames and VR QoS requirements.

As for the first strategy, modern Wi-Fi networks support both single and multi-user MIMO versions. With Single-User MIMO (SU-MIMO), an AP sends multiple spatial streams to a single user station (STA), which, in theory, increases data rate proportionally to the number of streams. However, the maximal number of spatial streams is usually limited by the number of STA antennas because of the small sizes of user devices. In contrast, with Multi-User MIMO (MU-MIMO), the AP can transmit spatial streams simultaneously to different STAs and enhance the entire network’s capacity. While MIMO seems to provide a significant gain [8], it comes with several challenges.

In particular, to use MIMO transmissions, an AP needs Channel State Information (CSI), which is obtained during the explicit sounding procedure. In this procedure, the AP transmits a Null Data Packet (NDP), with which STAs can estimate the channel between the AP and itself. After that, each STA compresses the channel, forms a Compressed Beamforming Frame (CBF), and sends it to the AP. The total duration of the procedure strongly depends on the number of STAs, the number of antennas at each device, and the MCS used for CBF transmission in the uplink. As a result, on the one hand, the sounding procedure induces a notable protocol overhead and, therefore, should not occur too frequently [9]. On the other hand, CSI becomes irrelevant with time, and data rates of MIMO transmissions decrease [10]. This effect is called channel aging [11] and is principally driven by mobility-induced Doppler spread, significant feedback delays, and alterations in the polarization characteristics of the propagation channel between channel estimation and data transmission.

State-of-the-art Wi-Fi 7 and 8 standards support up to eight antennas at the AP. During the standardization process, the Wi-Fi developers considered increasing the number of AP antennas to 16, but due to significant sounding overhead, this idea was postponed to the next generations of the standard. In this paper, we consider up to 16 antennas at the AP to estimate how the performance of the future Wi-Fi generations will be scaled.

The second strategy for increasing the QoS of VR traffic is delay-aware scheduling, which prioritizes frames nearing their delay threshold to meet QoS requirements. This approach is referenced in the literature, usually in the context of cellular networks, and employs various delay-aware scheduling metrics to optimize the performance. As highlighted in [12], the most common metrics are the Modified Largest Weighted Delay First (M-LWDF), the Exponential rule (EXP-rule), and the Logarithmic rule (LOG-rule). They differ in their sensitivity to packet delay, and their effectiveness may vary in different conditions. However, their performance in Wi-Fi MU-MIMO scenarios has not been carefully studied yet.

Finally, the network performance can be improved using recently proposed sensing-assisted communication [13], advanced antenna designs that take into account polarization [14,15], and UAV-assisted transmission methods that take into account antenna orientation and polarization [16]. Despite potentially providing performance improvements, they are not supported by the Wi-Fi standard yet, which is why they are considered out of scope of this research.

In our study, we examine the impact of increasing the number of antennas and using delay-aware schedulers at the AP on the performance of the future Wi-Fi networks serving VR traffic. This work is motivated by the observation that existing studies devoted to MU-MIMO rarely consider VR or any other delay-sensitive traffic [17,18,19]. At the same time, in works on VR, Wi-Fi MU-MIMO peculiarities are oversimplified or completely omitted [7,20], despite the induced overhead, channel aging, and other MIMO-related effects that may completely degrade the performance of the delay-sensitive VR traffic. In our work, we fill this gap by considering a Wi-Fi network with MU-MIMO capabilities, taking into account CSI overhead, channel aging, and VR traffic features. In particular, the contribution of this paper is as follows:

1. We built an ns-3-based simulation framework (the framework can be requested at https://wnlab.ru/wifi-mumimo-simulator, accessed on 27 August 2025) to evaluate Wi-Fi performance in VR scenarios with MU-MIMO support and the ability to use up to 16 antennas/spatial streams at the AP, which is not supported yet by the current Wi-Fi standard. The framework allows modeling explicit sounding procedure and channel aging, includes a realistic VR application model [21] and different delay-aware schedulers [12].
2. We determined the maximal number of VR users that can be served in a Wi-Fi MU-MIMO network in different scenarios. We analyzed how system performance depend on different factors, including the number of AP and STA antennas, different fading parameters, schedulers, and UL MCS selection algorithms used for CBF transmission.
3. We found and explained several effects that impact Wi-Fi performance when serving VR traffic:
  (a) In some scenarios with a highly dynamic environment, Wi-Fi MU-MIMO performance is not sensitive to the sounding period, i.e., AP can perform the sounding procedure very rarely (more than 100 ms), because the system performance is determined by the efficiency of the STA equalizers (see Section 4.2). Moreover, in this case, the performance of the SU-MIMO-only capable AP is very close to that of the MU-MIMO capable one (see Section 4.6).
  (b) In contrast to naive customer expectations, doubling the number of AP antennas does not usually double the network performance (see Section 4.3). The results reveal a critical finding: Scalability is not linear. In particular, doubling AP antennas from 8 to 16 yields only a 35% gain in capacity under typical conditions, which is well below 100% of the linear scaling one might expect. Moreover, when the duration of the sounding procedure is high due to low MCS used for BFR transmission, the rise of the number of AP antennas may even degrade the performance (see Section 4.8).
  (c) We highlight the importance of delay-aware scheduling approaches, which can provide a performance gain of up to 50%. Moreover, we show that in some scenarios the performance gain from doubling the number of antennas can be lower than from the implementation of a delay-aware scheduler (see Section 4.3).

The rest of this paper is organized as follows. Section 2 contains a review of the related works. Section 3 describes the system model, including the considered scenario, Wi-Fi MIMO operation, and scheduler details. In Section 4, we present the obtained numerical results. Section 5 concludes the paper.

## 2. Related Works

Usually, the literature explores the possibility of increasing the number of access point antennas without taking into account Wi-Fi protocol peculiarities or QoS requirements of the served traffic. For example, in [22], the authors demonstrated that it is advantageous to increase the number of antennas at the cellular base station from the point of view of downlink transmission signal-to-noise Ratio (SNR). However, they did not take into account the effect of the channel aging and protocol overhead. The study [23] investigated the performance of the cellular massive MIMO system in the uplink. Based on SNRs, the authors determined the required number of AP antennas to achieve a specified throughput.

In Wi-Fi, MIMO was initially introduced in the IEEE 802.11n amendment [24] and supported only single-user downlink transmissions with up to four spatial streams. With the advent of the 802.11ac/ax amendments, it has progressed to allow eight-stream multi-user transmissions both in downlink and uplink [9,25]. Currently, the topic of increasing the maximal number of antennas to 16 has been under discussion for quite some time as part of ongoing enhancements [17,18]. In [19], the authors examine the signal-to-noise ratio (SNR) required to obtain a particular packet error rate with 8 and 16 antennas. Similarly, in [26], the potential benefits of supporting 16 AP antennas alongside other 802.11be enhancements are explored. Specifically, the authors demonstrate that 802.11be achieves significant throughput improvements compared to 802.11ax, attributed to larger transmission bandwidths and advanced spatial multiplexing capabilities. However, they note that the throughput gains with 802.11be in realistic scenarios do not always correspond to the maximal data rate defined in the standard. For instance, factors such as station mobility [27] and transmission distance can contribute to deviations from the theoretical expectations. However, the aforementioned papers do not take into account the QoS of served traffic and/or channel aging.

As mentioned in Section 1, an increase in the number of AP antennas results in significant CSI overhead. Various solutions have been proposed to mitigate this problem. For example, in [28], the authors suggested a dimension reduction scheme to lower computational complexity and CSI overhead. The authors of [29] proposed to take into account the correlation of CSI in the frequency and time domains to efficiently compress CBF using a second derivative method. Another approach to reduce overhead is discussed in [18,30], where the authors explore the implicit sounding procedure, requiring less time compared to the explicit one. Additionally, the authors of [31] have developed an unsupervised learning-based method to shorten the sounding duration in Wi-Fi MIMO and increase network throughput. However, methods of CSI acquisition other than the explicit sounding procedure have not yet been standardized for use in modern Wi-Fi networks and cannot be used due to backward compatibility problems.

As for delay-aware scheduling, the survey [12] considers different types of schedulers in orthogonal frequency division multiple access networks. In particular, schedulers that take into account channel knowledge and knowledge of traffic types with corresponding QoS requirements are considered. For example, the LOG-rule scheduler provides the ability to prioritize the sending of packets for which the maximum delay time is close to expiration. In [32], additional schedulers are proposed to serve multiple types of traffic simultaneously. The authors propose a classification of scheduling algorithms and two new schedulers, Queue-HoL-M-LWDF and Modified-EXP-rule, that provide a balanced QoS among STAs with different types of traffic, while improving performance in terms of throughput, packet loss ratio, spectral efficiency, and fairness. However, these schedulers were applied within OFDMA technology in cellular systems; their efficiency in Wi-Fi MU-MIMO networks serving VR traffic has not been carefully studied yet.

Several studies have concentrated on scheduling strategies for Wi-Fi networks. In particular, the study [33] focuses on schedulers for uplink transmissions in Wi-Fi. The authors propose a resource unit allocation strategy that notably increases the goodput and reduces data transmission time in a single antenna Wi-Fi network. In [34], authors propose an algorithm that schedules STAs or STA groups for downlink transmissions using OFDMA resource units in 802.11ax networks. However, neither papers consider the delay-sensitive traffic.

In [35], the authors propose a LAST-PQ solution to support VR flows at the Wi-Fi AP. They implemented it in the driver of the off-the-shelf 802.11n AP with three antennas and showed that it allows reducing the latency up to 80%. However, the performance of this solution in the case of state-of-the-art APs with a higher number of antennas and MU-MIMO support is unclear.

In [7], the authors experimentally studied the performance of the state-of-the-art Wi-Fi 6 (IEEE 802.11ax) AP serving VR traffic of the Meta Quest 2 HMD. Surprisingly, they showed that the usage of the latest Wi-Fi features, such as OFDMA and MU-MIMO, decreases the QoS of the VR traffic. It can be explained by the high channel access delay induced by the scheduled access implemented in the considered AP in the case of enabling OFDMA and MU-MIMO features. This study highlights the need for improving the performance of state-of-the-art APs when serving VR traffic.

In contrast to previous research, in this paper, we develop a detailed simulation framework to evaluate the efficiency of Wi-Fi MU-MIMO networks serving VR traffic. We investigate the influence of different factors on the Wi-Fi performance, and show how it is scaled with the rise of the number of AP antennas in different conditions.

## 3. System Model

In this section, we describe a developed simulation framework for the estimation of the efficiency of Wi-Fi MU-MIMO networks in VR scenarios. In particular, we consider an IEEE 802.11be network operating in half-duplex mode with a single Access Point (AP) with $N_{tx}$ antennas and *M* STAs with $N_{rx}$ antennas each. STAs are distributed uniformly within a circle with radius *R* around the AP.

The AP is connected to the VR server using a wired high-speed connection and transmits VR flows with a given average bitrate to VR clients located at STAs.

Next, we provide a more detailed description of MIMO operation in Wi-Fi in Section 3.1, and the considered MU-MIMO scheduler in Section 3.2.

### 3.1. Wi-Fi MIMO Operation

To support MIMO and estimate the channel between itself and STAs, an AP uses the explicit sounding procedure [30]. To initiate the procedure, the AP selects STAs, the CSI of which it wants to update, and sends a Null Data Packet Announcement (NDPA) frame to them. NDPA contains the CSI parameters for each STA. After a short interframe interval (SIFS) following the NDPA, the AP sends an NDP frame. Having received the NDP frame, each STA estimates the channel between the AP and itself using the dedicated part of the NDP preamble. After that, the AP sequentially requests CSI from the STAs by sending a Beamforming report poll (BFRP) frame, in response to which the intended STA sends back a CBF. The AP can use more complicated BFR collection techniques using OFDMA [36] or UL MU-MIMO to reduce the procedure overhead. However, they require an accurate estimation of the uplink channel quality, so we do not consider them in the current paper.

Similar to [37], we assume that the AP initiates the explicit sounding procedure periodically every *T* milliseconds.

For each data transmission, the AP selects the set of intended STAs and the number of spatial streams (NSS) assigned for each STA. We introduce the NSS vector as

(1) $$S=\left[s_{1},s_{2},\ldots ,s_{i},\ldots ,s_{M}\right],$$

where $s_{i}$ represents the NSS assigned to STA *i*. We assume that $s_{i}>0$ only if the AP has data for STA *i*. The received signal vector $y_{k}$, after receiver processing at the STA *k* at a given subcarrier, can be represented as:

(2) $$y_{k}=W_{k}\left[H_{k}P_{k}x_{k}+\sum_{i,i\neq k}H_{k}P_{i}x_{i}+n_{k}\right],$$

where

- $x_{k}$ ($x_{i}$) represents the $s_{k}\times 1$ ($s_{i}\times 1$) transmitting signal vector intended for STA *k* (STA *i*),
- $H_{k}$ is the $N_{rx}\times N_{tx}$ channel matrix between the AP and STA *k*,
- $P_{k}$ ($P_{i}$) is the $N_{tx}\times s_{k}$ ($N_{tx}\times s_{i}$) precoding matrix,
- $W_{k}$ is the $s_{k}\times N_{rx}$ detection matrix,
- $n_{k}$ is $N_{rx}\times 1$ noise vector.

We employ a Zero-Forcing (ZF) [8] precoder at the transmitter and the Minimum Mean Square Error (MMSE) [19] equalizer (detection matrix) at the receiver. These methods are chosen for their widespread usage [38,39]. In the case of perfect CSI, ZF cancels multi-user interference at the intended STAs. At the same time, the MMSE equalizer processes the received signal to minimize residual error in case of imperfect CSI or channel aging. For mapping SNR to packet loss rate, we use the default ns-3 NIST error rate model.

For MIMO channel modeling, we consider far-field conditions and use the TGax channel model [40] recommended by the IEEE 802.11 Task group for the performance evaluation of Wi-Fi networks. The TGax channel model is a comprehensive model designed to simulate the key effects that a wireless signal experiences when propagating through indoor and outdoor environments. It is a Geometry-based Stochastic Channel Model (GSCM) that uses a statistical representation of the environment based on scattering clusters. When generating a MIMO channel, it takes into account multipath propagation, angular and delay spread, frequency-selective and time-varying fading, and antenna polarization in a statistical manner, which is practical for system-level simulations. In our experiments, we use the Model-B and Model-D delay profiles, which correspond to the indoor residential and office cases. Also, we vary the environmental (scatterers) speed *E* that determines how quickly the channel changes over time.

To study the impact of MU-MIMO on the performance of Wi-Fi networks serving VR traffic without side effects caused by the non-ideality of MCS selection algorithms, e.g., [41,42,43], we consider that the AP uses ideal rate adaptation in the downlink. In particular, we assume that the AP can precisely estimate the achieved SNR for every spatial stream in every transmission and select the fastest MCS that provides a packet loss ratio below ϵ. However, to take into account the negative impact of the CSI aging effect on the MIMO performance, we assume that the MIMO precoder is constructed based on the last obtained CSI, and, as a result, its efficiency degrades with the time passed from the last sounding procedure.

For uplink MCS selection, we also consider the ideal MCS selection algorithm for BFR transmission. However, in Section 4.8, we compare the performance of the ideal UL MCS selection algorithm with that of more conservative ones.

### 3.2. Wi-Fi MIMO Scheduler

To solve the problem of STA and NSS selection, for each NSS vector S, we determine the utility U(S) of the corresponding transmission calculated as follows

(3) $$U\left(S\right)=\sum_{i=1}^{M}G_{i}\left(S\right),$$

where $G_{i}\left(S\right)$ is the scheduling weight of STA *i* in the vector S. $G_{i}\left(S\right)$ depends on the selected scheduling metric.

Hence, the problem can be formulated as:

(4) $$\max_{S\in \Omega }U\left(S\right)=\max_{S\in \Omega }\sum_{i=1}^{M}G_{i}\left(S\right),$$

where Ω is a set of NSS vectors obeying the following requirements:

(5) $$\begin{array}{cc} \; & \forall S\in \Omega ,\forall s_{i}\in S\Rightarrow s_{i}\leq N_{rx};\\ \; & \forall S\in \Omega \Rightarrow \sum_{i=1}^{M}s_{i}\leq N_{tx}. \end{array}$$

To solve (4), in this paper, we develop a greedy algorithm to find the NSS vector for transmission. According to this algorithm, the AP starts from an empty NSS vector ${\tilde{S}}_{0}$. At step *i*, AP finds a vector ${\tilde{S}}_{i}$ such that

(6) $${\tilde{S}}_{i}=\underset{S\in \Omega ({\tilde{S}}_{i-1})}{arg max}U\left(S\right),$$

where $\Omega ({\tilde{S}}_{i-1})\subset \Omega$ is a set of NSS vectors that can be constructed from ${\tilde{S}}_{i-1}$ by incrementing by one the NSS value corresponding to exactly one STA. If ${\tilde{S}}_{i}$ is not unique, i.e., several vectors have the same utility, the AP chooses one of them randomly. Next, if $U\left({\tilde{S}}_{i}\right)<U\left({\tilde{S}}_{i-1}\right)$, the AP selects vector ${\tilde{S}}_{i-1}$ for transmission. If $U\left({\tilde{S}}_{i}\right)\geq U\left({\tilde{S}}_{i-1}\right)$, it selects for transmission ${\tilde{S}}_{i}$ if the total number of spatial streams in ${\tilde{S}}_{i}$ equals $N_{tx}$. Otherwise, if $U\left({\tilde{S}}_{i}\right)\geq U\left({\tilde{S}}_{i-1}\right)$ and the total number of spatial streams is less that $N_{tx}$, the AP proceeds to the next step and finds ${\tilde{S}}_{i+1}$. Note that we can simulate an SU-MIMO-only capable AP using this approach (see Section 4.6), if we additionally add the constraint that vectors in $\Omega ({\tilde{S}}_{i-1})$ shall have only a single non-zero element.

The most widely used scheduling metric in wireless networks is called Proportional Fair (PF). PF tries to maximize the geometric average throughput of the network to balance between spectral efficiency and fairness. PF metric $G_{i}^{\mathrm{PF}}\left(S\right)$ for STA *i* in NSS vector S is calculated as follows:

(7) $$G_{i}^{\mathrm{PF}}\left(S\right)=\frac{d_{i}\left(S\right)}{D_{i}},$$

where $d_{i}\left(S\right)$ is the data rate of STA *i* in a data transmission with NSS vector S, and $D_{i}$ is the average data rate of STA *i*. This metric is efficient for handling traffic that does not require low latency [32].

A number of scheduling metrics for serving delay-sensitive traffic were proposed in the literature [32]. The common approach is to multiply the PF metric (7) by some function that takes into account the delay of the Head-of-Line (HoL) packet. Next, we describe three popular approaches for scheduling delay-sensitive traffic.

The most well-known metric for handling delay-sensitive traffic is M-LWDF. This metric increases linearly with the current HoL packet delay. As a result, M-LWDF metric $G_{i}^{M\text{-}\mathrm{LWDF}}\left(S\right)$ for STA *i* in NSS vector S is defined as follows:

(8) $$G_{i}^{M\text{-}\mathrm{LWDF}}\left(S\right)=\frac{-\log\delta_{i}}{\tau_{i}}\cdot t_{i}\cdot G_{i}^{\mathrm{PF}}\left(S\right)=Q_{i}\cdot t_{i}\cdot G_{i}^{\mathrm{PF}}\left(S\right),$$

where $t_{i}$ is the delay of the HoL packet for STA *i*, $\tau_{i}$ is the target delay, $\delta_{i}$ is the target probability that the HoL packet delay exceeds the target delay, and $Q_{i}=\frac{-\log\delta_{i}}{\tau_{i}}$.

The second metric to be explored is called the EXP-rule. In contrast to M-LWDF, the EXP-rule exponentially increases the priority of STA *i* with its HoL packet delay. EXP-rule metric $G_{i}^{\mathrm{EXP}}\left(S\right)$ can be calculated as follows:

(9) $$G_{i}^{\mathrm{EXP}}\left(S\right)=\exp\left(\frac{Q_{i}\cdot t_{i}}{1+\sqrt{h}}\right)\cdot G_{i}^{\mathrm{PF}}\left(S\right),$$

where *h* takes into account the average delay for all STAs as follows:

(10) $$h=\frac{1}{M_{cur}}\sum_{i=1}^{N}t_{i},$$

where $M_{cur}$ is the total number of STAs for which the AP currently has buffered packets, and $t_{i}=0$ if the AP does not have data for transmission to STA *i*.

The last metric discussed in the current paper is the LOG-rule. Unlike the EXP-rule, it increases the priority of STA *i* proportionally to the logarithm of the HoL packet delay. LOG-rule metric $G_{i}^{\mathrm{LOG}}\left(S\right)$ is calculated as follows:

(11) $$G_{i}^{\mathrm{LOG}}\left(S\right)=\ln\left(1+Q_{i}\cdot t_{i}\right)\cdot G_{i}^{\mathrm{PF}}\left(S\right).$$

In this paper, we compare the performance of the aforementioned metrics when serving VR traffic in Wi-Fi MU-MIMO networks.

## 4. Numerical Results

In this section, we investigate the maximal Wi-Fi MIMO performance when serving VR traffic in different scenarios depending on various factors. First, in Section 4.1, we describe VR application traffic generation and main KPIs. Next, in Section 4.2, we evaluate the impact of the sounding period. Section 4.3 and Section 4.4 present how the Wi-Fi performance changes with the increase of the number of antennas at the AP and STAs. The impact of the channel model is investigated in Section 4.5. Next, in Section 4.6, we show how the Wi-Fi performance depends on the MU-MIMO support. Section 4.7 presents the results for different downlink MCS selection algorithms. Finally, in Section 4.8, we highlight how the performance is affected by the MCS used in the sounding procedure to collect CBF.

Unless otherwise stated, we use the default simulation parameter values presented in Table 1.

### 4.1. VR Application and Considered KPIs

Different VR traffic modeling approaches were considered recently [5,21,44,45,46,47,48]. In this paper, we use the VR traffic generation model proposed in [21]. The model accurately reproduces the behavior of the off-the-shelf Pico Neo 2 VR headset [49].

The current paper is mainly focused on the performance of the Wi-Fi MU-MIMO in the downlink. In the downlink, the considered application transmits two UDP flows with video streams intended for each eye. Each stream consists of periodically generated frames with a frequency of 72 frames per second (FPS) and an average bitrate of 40 Mbps, resulting in 144 FPS and 80 Mbps in total. Frame sizes are taken from the database with real video streams encoded using NVIDIA NVENC encoder [50] with ultra-low latency preset [21] and depend on the VR content. Streams for different eyes are slightly shifted to reproduce the behavior of the VR testbed.

For simplicity, we do not consider VR application uplink streams that carry the information about headset and controller positions. The total average bitrate of these streams is low (less than 1 Mbps [21]) compared to the downlink streams. As a result, they can be easily served using either uplink OFDMA transmissions in the downlink TXOPs, or the separate link in the multi-link IEEE 802.11be VR devices.

To take into account deterministic requirements of delay-sensitive VR traffic, we consider that a VR frame is lost (or corrupted) if at least one of its packets is not received before the delay budget $\tau_{\max}$. Next, we consider that the QoS requirements of a STA are satisfied if the percentage of lost VR frames in the experiment does not exceed $p_{\max}$. Similar to [4,5,6], we consider $p_{\max}=1\%$ and $\tau_{\max}=20$ ms in our simulation.

To evaluate the system performance taking into account the deterministic delay target of VR traffic, we estimate the number $M_{sat}\left(M,N_{tx},N_{rx},E,T,R\right)$ of satisfied STAs in a given scenario, which is defined by the number *M* of STAs in the network, the number of antennas at the AP ($N_{tx}$) and STAs ($N_{rx}$), environmental speed *E*, sounding period *T*, and network radius *R*. After that, we find the maximum number *V* of satisfied STAs over different number *m* of STAs:

(12) $$V=\max_{m}M_{sat}\left(m,N_{tx},N_{rx},E,T,R\right).$$

Since the sounding period significantly impacts the system performance and can be changed by the AP, we also estimate the maximal number $V_{\max}$ of satisfied STAs over different number *m* of STAs and sounding period *t*:

(13) $$V_{\max}=\max_{m,t}M_{sat}\left(m,N_{tx},N_{rx},E,t,R\right).$$

### 4.2. Impact of Sounding Period

**Figure 1:**
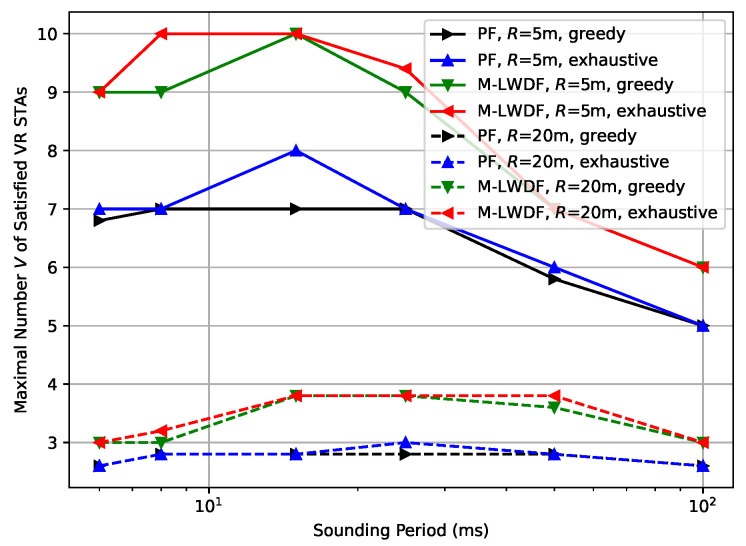
Maximal number *V* of the satisfied VR STAs depending on the sounding period for different Wi-Fi MU-MIMO schedulers and network radius.

First of all, we estimate the efficiency of the developed greedy scheduling algorithm compared to the exhaustive search algorithms. Figure 1 shows the results for the case of $N_{tx}=8$ antennas at the AP, $N_{rx}=2$ antennas at STAs, and low environmental speed (E=0.089 kmph) for different schedulers and network radius *R*. As can be seen, the results of both algorithms in terms of achieving the maximal VR capacity are very close or even coincide. Since even in this simple scenario the exhaustive search algorithm is extremely computationally complex, in all following experiments, we will provide only the results for the greedy algorithm.

**Figure 2:**
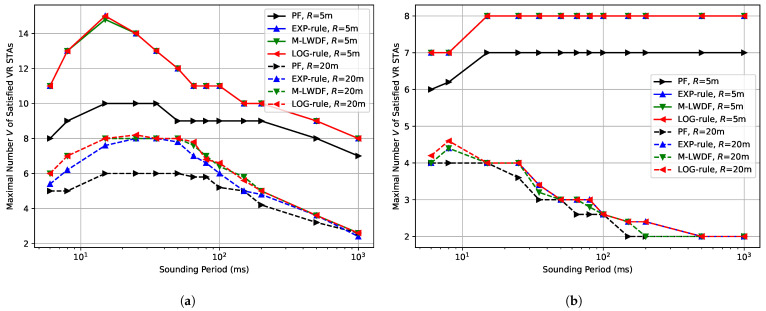
Maximal number *V* of the satisfied VR STAs depending on the sounding period in the case of $N_{tx}=16$ antennas at the AP and (**a**) E=0.089 kmph, (**b**) E=5 kmph.

Next, we estimate the maximal number *V* of VR STAs that can be satisfied for a given sounding period, which is defined by (12). Figure 2a shows the results for the case of $N_{tx}=16$ antennas at the AP and low environmental speed (E=0.089 kmph) for different schedulers and network radius *R*.

The behavior of all curves is determined by the following two factors. When the sounding period is short, the share of channel resources spent on the sounding procedure is significant, and Wi-Fi performance is poor. Moreover, it should be mentioned that too frequent sounding procedure may increase power consumption of user devices, however, this issue is out of scope of the current paper. After reaching their maximum values, all curves start to fall because the channel information becomes outdated and the efficiency of MIMO precoding decreases, which reduces transmission data rates.

Notably, all considered delay-aware schedulers (M-LWDF, LOG-rule, and EXP-rule) always show very close performance. It can be explained by the fact that we do not consider non-VR traffic in our scenarios, and all VR flows have the same QoS requirements. However, they significantly outperform the PF scheduler (up to 50% for 15 ms sounding period and R=5 m), which highlights the importance of using a delay-aware scheduling approach when serving VR traffic. It happens because the PF scheduler decreases the service rate of VR STAs with poor channel conditions, and they become unsatisfied. At the same time, delay-aware schedulers are trying to serve all VR STAs without violating delay thresholds, and, as a result, the maximum number of satisfied VR STAs is higher. The difference between PF and delay-aware schedulers is higher in the case of higher absolute numbers of satisfied VR STAs, e.g., in the case of R=5 m compared to R=20 m.

Figure 2b shows the results for the same experiment, but for high environmental speed (E=5 kmph). In general, the results for R=20 m are similar to the ones for low environmental speed. The maximal number of satisfied VR STAs is decreased because of the lower performance of MU-MIMO precoding due to faster channel fading. In this case, the optimal sounding period becomes shorter than 10 ms, i.e., the AP should perform the channel sounding procedure almost before each MU-MIMO transmission, which significantly increases protocol overhead.

In contrast to the previously described cases, the behavior for R=5 m and high environmental speed is completely different. After the initial rise that can be explained by the decrease of sounding overhead, we can see a floor, i.e., the network performance seems not to be affected by the aging of channel information.

To explain this effect, we conduct the following experiment. For each combination $\left(N_{tx},R,E\right)$, we find the maximal number of satisfied VR STAs $M=V_{\max}$. After that, we set a 100ms sounding period and run the experiment to gather the statistics of scheduling decisions to estimate how the network performance depends on the age of channel information (i.e., the time since the last channel information update) for a given combination $\left(N_{tx},R,E,M\right)$.

**Figure 3:**
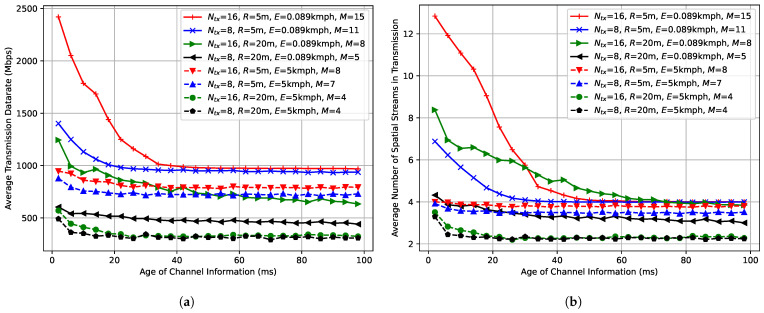
Impact of the age of channel information on: (**a**) average transmission data rate, (**b**) average number of spatial streams.

Figure 3a shows the average transmission data rate depending on the age of channel information. As can be expected, channel information aging decreases the average data rate because MIMO precoding becomes less accurate and, as a result, the achieved SNR decreases. However, all curves tend to some data rate floor with the rise of the age of channel information. This floor for every configuration is different and is defined by the performance of STAs’ equalizers in a given scenario. Performance of the equalizer does not depend on the age of channel information at the AP, because STAs estimate the channel for equalization using the preamble of the received transmission.

In most cases, e.g., when the environmental speed is low, the data rate achieved with fresh channel information is significantly higher than the corresponding data rate floor, and, as a result, it is beneficial to perform the sounding procedure frequently. In contrast, for E=5 kmph and R=5 m datarate floor and datarate after right after sounding procedure are very close, i.e., the channel information becomes irrelevant very quickly. Additionally, the advantage of fresh channel information is diminished by the sounding procedure overhead.

Figure 3b shows the average number of spatial streams depending on the age of channel information. It can be seen that when operating at the data rate floor, the AP uses no more than four spatial streams, which equals the number of STA antennas in our simulation. This additionally proves that in the data rate floor region, the network performance is defined by the equalizer, which works better when the total number of transmitted spatial streams is fewer than the number of receiving antennas of each STA.

***Lessons learned:*** In most cases, the sounding period significantly impacts the VR performance in Wi-Fi MU-MIMO networks. This behavior can be explained by the influence of the sounding procedure overhead at short periods and the precoder aging at long periods. Despite a similar effect being observed for full buffer traffic, in VR scenarios, the impact is more pronounced. It happens because VR traffic demands a stable data rate due to the strict delay requirements, and the precoder aging may increase delays and, as a consequence, VR frames drop more probably at the end of the sounding period.

However, in rare cases, e.g., for high environmental speed and short network radius, we do not observe any impact of precoder aging. It happens because the network operates at a data rate floor region, where the performance mainly depends on the STA equalizers, while the AP precoder ages very quickly. As a result, the AP can use a rather long sounding period without affecting VR performance.

The difference between the performance of different delay-aware schedulers is rather negligible, but, as can be expected, they notably outperform the PF scheduler, and the gain increases (up to 50%) with the number of STAs satisfied by the PF scheduler.

### 4.3. Impact of Number of Antennas at the AP

Let us turn to the question of how the Wi-Fi performance is scaled with the number of AP antennas. In general, the expectation of a naive Wi-Fi customer is that the number of satisfied VR STAs should change proportionally to the number of AP transmitting antennas, because APs can send more spatial streams. However, in practice, the gain is lower due to several factors, which we discuss in this section.

**Figure 4:**
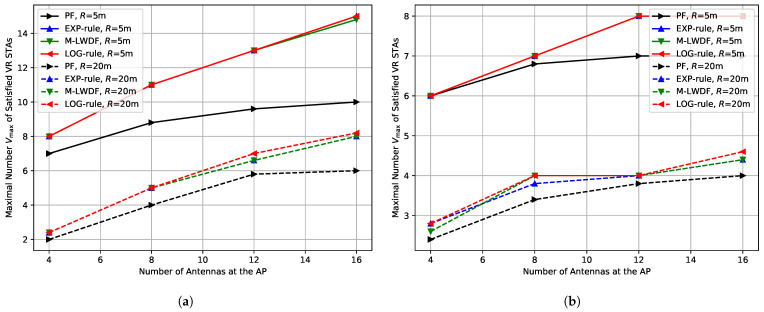
Maximal number $V_{\max}$ of the satisfied VR STAs depending on the number of antennas at the AP in the case of (**a**) E=0.089 kmph, (**b**) E=5 kmph.

Figure 4a shows the maximal number $V_{\max}$ of satisfied VR STAs defined by (13) depending on the number of antennas at the AP for low environmental speed (E=0.089 kmph).

For delay-aware schedulers and short network radius (R=5 m), doubling the number of antennas increases the number of satisfied VR STAs by only about 35%. It can be explained by the following factors. First, the total maximum transmit power of a Wi-Fi device is fixed and distributed between all spatial streams. As a result, the average signal power of a stream decreases when we increment the number of streams in transmission. Second, in the case of imperfect (aged) channel information, the ZF precoder does not fully eliminate inter-stream interference, and its amount increases with the number of transmitted streams. As a result, in case of a high number of antennas, it is rather difficult to efficiently use the maximal number of spatial streams even if the channel information is rather fresh (e.g., compare leftmost values of blue and red solid lines in Figure 3b).

At the same time, the performance of Wi-Fi with the PF scheduler is worse, and changing the number of antennas from 8 to 16 adds only one satisfied VR STA. Again, it is explained by the deprioritization of VR STAs with poor channel conditions. Moreover, it is interesting that if we consider some baseline system configuration with the PF scheduler, in some cases it is more profitable to implement the delay-aware scheduler than to double the number of antennas, e.g., when we have an AP with 8 antennas and a scenario with low environmental speed and short network radius.

For a high network radius (R=20 m), the relative gain is higher (60–100% for delay-aware schedulers), because the impact of inter-stream interference is lower due to the higher channel noise level and lower total number of transmitted spatial streams.

High environmental speed (E=5 kmph) leads to a decrease in the maximal number of satisfied VR STAs, see Figure 4b. Moreover, in some cases, there is no gain at all. For example, when we increase the number of antennas from 12 to 16 for a short network radius (R=5 m).

***Lessons learned:*** In many cases, Wi-Fi performance is not scaled linearly with the increase in the number of AP antennas. It happens due to several factors, including the power limitation, inter-stream interference caused by the precoder aging, and sounding overhead increase. Moreover, in some cases, it is more profitable to implement the delay-aware scheduler than to double the number of antennas.

### 4.4. Impact of the Number of Antennas at STA

**Figure 5:**
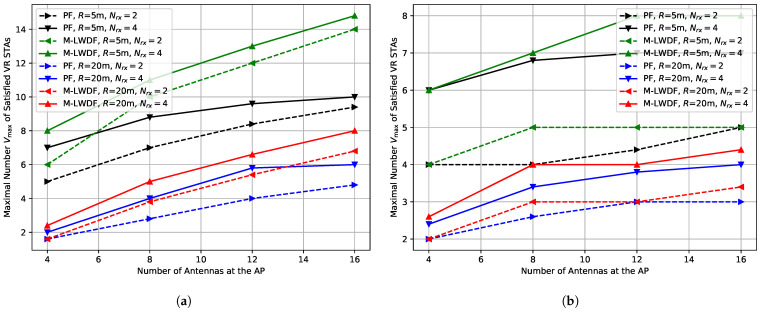
Impact of number of receiving antennas on the maximal number $V_{\max}$ of the satisfied VR STAs depending on the number of antennas at the AP in the case of (**a**) E=0.089 kmph, (**b**) E=5 kmph.

Let us compare how the Wi-Fi performance depends on the number $N_{rx}$ of STA antennas. Figure 5a shows the comparison of the Wi-Fi configurations with $N_{rx}=2$ and $N_{rx}=4$ for the case of low environmental speed (E=0.089 kmph). To limit the number of compared solutions, we do not show the curves for the LOG-rule and EXP-rule schedulers, because they provide results almost identical to those of M-LWDF.

In all considered cases, the results for two and four STA antennas are rather close, especially for the M-LWDF scheduler. Moreover, the difference between network configurations is decreasing with the rise in the number of AP antennas. It can be explained by the fact that the impact of AP precoding on network performance becomes dominant, whereas the lower performance of equalizers due to a lower number of STA antennas is almost compensated by the lower sounding procedure overhead.

Figure 5b shows the comparison of the Wi-Fi configurations with two and four STA antennas for the case of high environmental speed (E=5 kmph). Due to faster channel aging, the efficiency of AP precoding decreases, and the impact of STA equalizers becomes higher. As a result, the difference in network performance is higher in this case, and the decrease in the number of STA antennas from four to two may decrease the maximal number of satisfied VR STAs up to 40%.

***Lessons learned:*** In the majority of cases, the number of STA antennas does not significantly change the VR performance, because it usually depends on the precoder efficiency determined by the number of AP antennas. In contrast, when the network operates at the data rate floor (see Section 4.2), VR performance is significantly increased (up to 50%) when we change the number of antennas from two to four due to the higher efficiency of the STA equalizers.

### 4.5. Impact of the Channel Model

**Figure 6:**
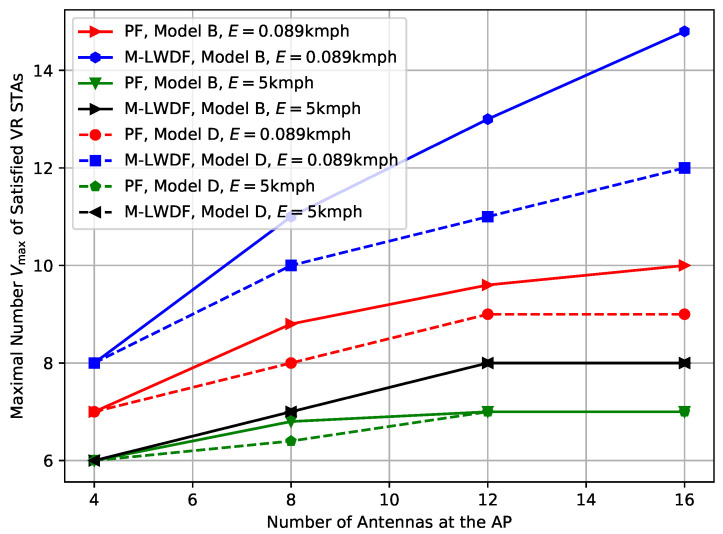
Impact of the channel model on the maximal number $V_{\max}$ of the satisfied VR STAs depending on the number of antennas at the AP in the case of R=5 m.

Now, let us consider the impact of the channel model on the Wi-Fi performance. Figure 6 shows the results for the case of R=5 m and two TGax channel models with different delay spreads: Model-B corresponding to the residential environment, and Model-D corresponding to the office environment.

As we can see, when the environmental speed is low (E=0.0089 kmph), the usage of Model-D results in a notable scaling decrease. It can be explained by the faster channel aging that happens due to higher delay spread and the number of environmental clusters. At the same time, the results for the high environmental speed (E=5 kmph) are almost identical for both channel models. Similar to the results obtained in the previous sections, it can be explained by the fact that systems performance is mostly determined by the STA equalizers, but not on the AP precoder.

***Lessons learned:*** In more complicated environments with higher delay spread, the performance scaling decreases due to faster channel aging.

### 4.6. Impact of MU-MIMO Operation

As we show in the previous sections, in many cases, Wi-Fi MU-MIMO performance scales slowly with the increase in the number of antennas at the AP. As a result, the question arises: Do we really need to use MU-MIMO in all cases, or does the usage of SU-MIMO show almost similar performance? SU-MIMO is known to be more robust to channel aging, requires much less frequent channel sounding, and, in general, the SU-MIMO-only implementation is simpler compared to the MU-MIMO one.

To answer this question, we estimate the maximal number $V_{\max}$ of satisfied VR STAs in the case when MU-MIMO is disabled, i.e., the AP considers only transmission configurations where all spatial streams are destined to exactly one STA when solving the scheduling problem (4).

**Figure 7:**
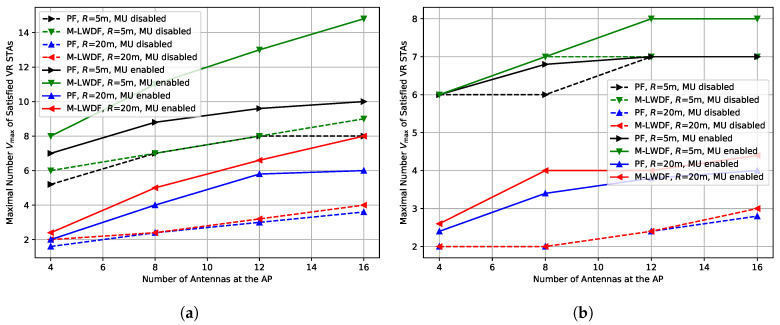
Impact of MU-MIMO operation on the maximal number $V_{\max}$ of the satisfied VR STAs depending on the number of antennas at the AP in the case of (**a**) E=0.089 kmph, (**b**) E=5 kmph.

The achieved results are shown in Figure 7. As we can see, in most cases, MU-MIMO significantly boosts the network performance even though it produces more sounding overhead. The exception is the case with high environmental speed (E=5 kmph) and short network radius (R=5 m). In this case, as we described earlier in Section 4.2, the system operates in the data rate floor region, and MU-MIMO and SU-MIMO transmissions with the same total number of spatial streams provide very similar capacity. As a result, we do not see any significant gain from the MU-MIMO in this case.

***Lessons learned:*** In the majority of cases, MU-MIMO notably improves Wi-Fi performance in VR scenarios compared to SU-MIMO due to a higher average number of streams in the transmissions. However, when the network operates at the data rate floor, the results of SU-MIMO and MU-MIMO are rather close, so in this case, SU-MIMO-only operation can be preferred due to simplicity.

### 4.7. Impact of Downlink MCS Selection Algorithm

**Figure 8:**
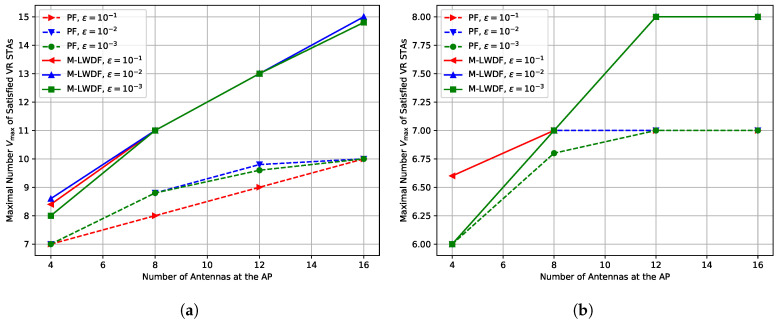
Impact of maximal packet loss ratio ϵ in the downlink MCS selection algorithm on the maximal number $V_{\max}$ of the satisfied VR STAs depending on the number of antennas at the AP for R=5 m in the case of (**a**) E=0.089 kmph and (**b**) E=5 kmph.

In this section, we examine the impact of the maximal packet loss ratio ϵ in the downlink MCS selection algorithm on the Wi-Fi performance, see Figure 8.

In general, the results for all considered ϵ values are rather close, because the unsuccessful transmission attempts do not significantly increase the delay of the VR frames. In contrast, the increase in ϵ may even lead to slightly better results, because in some cases, MCS selection algorithms choose faster MCSs.

***Lessons learned:*** The maximal packet loss ratio ϵ does not significantly affect the performance of the network, because the higher probability of errors is compensated by the selection of faster MCSs.

### 4.8. Impact of Uplink MCS Selection Algorithm

Finally, let us estimate the impact of the uplink MCS selection algorithm used in the sounding procedure for BFR transmission. In previous sections, we used the ideal MCS selection algorithm. In contrast, in several papers [30,51] it is supposed that the sounding procedure must be robust, so the most robust MCS0 with a single spatial stream must be used. Because of that, in this section, we compare the following solutions:

- *Ideal.* The dynamic MCS selection algorithm, according to which the AP selects the fastest MCS and the number of spatial streams for each STA, taking into account current channel conditions;
- *MCS0.* The constant MCS selection algorithm, which always uses the most robust MCS0 with a single spatial stream, similar to [30,51];
- *MCS7.* The constant MCS selection algorithm, which always selects MCS7 with a single spatial stream. It is selected as a compromise between the data rate and reliability, because MCS7 is enough to transmit BFRs reliably in all considered scenarios.

**Figure 9:**
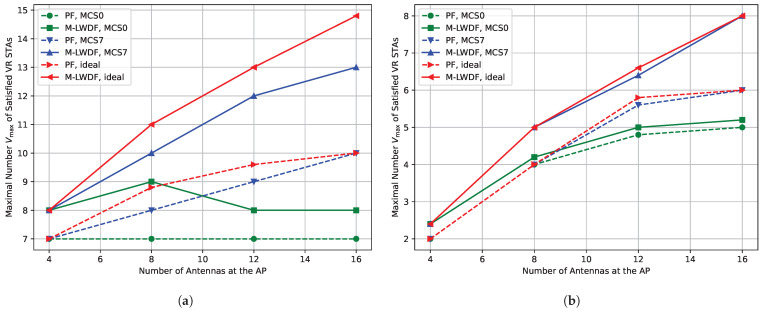
Impact of sounding procedure on the maximal number $V_{\max}$ of the satisfied VR STAs depending on the number of antennas at the AP for E=0.089 kmph in the case of (**a**) R=5 m and (**b**) R=20 m.

Figure 9a shows the dependence of the maximal number $V_{\max}$ of the satisfied VR STAs depending on the number of antennas for low environmental speed (E=0.089 kmph) and short network radius (R=5 m). The usage of MCS7 instead of ideal MCS decreases the maximal number of satisfied VR STAs up to 15%. In contrast, the usage of MCS0 significantly decreases the network performance. Moreover, it does not allow the number of satisfied VR STAs to rise when we increase the number of antennas at the AP from 4 to 16. It happens because the sounding procedure with MCS0 produces heavy overhead and becomes too long, which increases the optimal sounding period and decreases the average transmission data rate. Moreover, when the AP conducts a long sounding procedure, VR traffic is blocked and, as a result, VR frames may be dropped due to lifetime expiration.

Figure 9b shows the dependence of the maximal number $V_{\max}$ of the satisfied VR STAs depending on the number of antennas for low environmental speed (E=0.089 kmph) and large network radius (R=20 m). In this case, the ideal MCS selection algorithm has to use more robust MCSs, so the difference between the Ideal and MCS7 notably decreases. In contrast, MCS0 notably decreases the gain from increasing the number of AP antennas. Also, when MCS0 is used, we almost do not see the difference between PF and delay- aware schedulers.

***Lessons learned:*** MCS selection for BFR collection notably influences the achieved results. If the MCS selection algorithm is too conservative, it can completely block Wi-Fi performance scaling when we increase the number of AP antennas.

## 5. Conclusions

This study investigated the scalability of Wi-Fi networks under the stringent demands of multi-user Virtual Reality and Metaverse applications, challenging the conventional wisdom that simply adding more Access Point (AP) antennas is a panacea for performance. Through a purpose-built, high-fidelity ns-3 simulation framework supporting up to 16 antennas and realistic VR traffic models, we uncovered non-intuitive scalability limits and provided key design insights.

Using the framework, we reveal several key effects.

First, the performance of Wi-Fi MIMO in VR scenarios is not always sensitive to the explicit sounding period. We show that in cases with high environmental speed and short network radius, the network mostly operates in a data rate floor regime, where the performance depends only on the work of STA equalizers, but not on the AP precoding. Moreover, in this case, the performance of SU-MIMO only capable AP and MU-MIMO capable AP becomes very close, making SU-MIMO only operation a viable alternative due to its lower overhead, robustness, and simpler implementation.

Second, in contrast to possible expectations of Wi-Fi customers, which assume that increasing the number of AP antennas significantly improves performance in the majority of scenarios, the gains are often sublinear because of fixed total transmit power and inter-stream interference caused by channel aging. In particular, doubling AP antennas from 8 to 16 yields increases the number of satisfied VR users only by 35%, which is well below 100% that one might expect. Moreover, in high-mobility scenarios, additional AP antennas may even degrade the performance if the AP uses a conservative MCS selection algorithm in the sounding procedure.

Third, intelligent, delay-aware scheduling is a more potent tool than often realized. It can boost the number of satisfied users by up to 50%, which can surpass the benefit of doubling the number of AP antennas.

These findings highlight the limits of Wi-Fi performance when serving VR traffic. They show that increasing the Wi-Fi peak datarate by adding AP antennas may have a very limited impact on the network performance. Achieving scalability for VR over Wi-Fi requires a holistic approach that moves beyond a singular focus on peak data rates and takes into account spectral and energy efficiency of MU-MIMO in certain conditions, tracking of the antenna polarization. The interplay between physical hardware cost, algorithmic intelligence, and the wireless environment is paramount.

## Figures and Tables

**Table 1:** Default simulation parameters.

VR parameters	
VR average bitrate	80 Mbps
VR frame rate	144 FPS
VR video encoder	NVENC, ultra-low latency preset
VR delay constraint, $\tau_{\max}$	20 ms
VR maximal allowable percentage of corrupted frames, $p_{\max}$	1%
Scheduler parameters	
Target delay, $\tau_{i}$	20 ms
Target probability of exceeding the target delay, δ	0.01
Explicit sounding procedure parameters	
Subcarrier grouping	16-tone
Ψ angle bit depth	7 bits
Φ angle bit depth	9 bits
Wi-Fi MAC layer parameters	
Maximal duration of a frame sequence, $TXOP_{limit}$	5440 µs
Block ACK window size	1024
maximal packet loss ratio, ϵ	$10^{-3}$
Wi-Fi PHY layer parameters	
$N_{tx}$	16
$N_{rx}$	4
AP TX power	20 dBm
STA TX power	16 dBm
Noise level	−94 dBm for 20 MHz
Precoder	ZF
Equalizer	MMSE
Channel parameters	
Channel model	TGax Model-B
Bandwidth	40 MHz
Central carrier frequency	5.0 GHz
SNR to packet loss ratio model	NIST

## Data Availability

Data are contained within the article.

## Abbreviations

The following abbreviations are used in this manuscript:

AP	Access Point
CBF	Compressed Beamforming Frame
CSI	Channel State Information
FPS	Frames Per Second
HMD	Head-Mounted Display
HoL	Head-of-Line
MCS	Modulation and Coding Scheme
MMSE	Minimum Mean Square Error
MIMO	Multiple-Input Multiple-Output
MU-MIMO	Multi-User MIMO
M-LWDF	Modified Largest Weighted Delay First
NDP	Null Data Packet
NSS	Number of Spatial Streams
OFDMA	Orthogonal Frequency-Division Multiple Access
QoS	Quality of Service
SISO	Single-Input Single-Output
SNR	Signal-to-Noise Ratio
STA	Station
SU-MIMO	Single-User MIMO
VR	Virtual Reality
ZF	Zero Forcing
